# Isorhamnetin and anti-PD-L1 antibody dual-functional mesoporous silica nanoparticles improve tumor immune microenvironment and inhibit YY1-mediated tumor progression

**DOI:** 10.1186/s12951-023-01967-3

**Published:** 2023-07-05

**Authors:** Huijuan Liu, Jingxia Han, Ying Lv, Zihan Zhao, Shaoting Zheng, Yu Sun, Tao Sun

**Affiliations:** 1 State Key Laboratory of Medicinal Chemical Biology and College of Pharmacy, Nankai University, Tianjin, China; 2 Tianjin Key Laboratory of Early Druggability Evaluation of Innovative Drugs, Tianjin International Joint Academy of Biomedicine, Tianjin, China

**Keywords:** Epithelial–mesenchymal transformation (EMT), Ubiquitin-specifific protease 7 (USP7), Yin-yang-1 (YY1), Isorhamnetin, Anti-PD-L1 monoclonal antibody, Mesoporous silica nanoparticles

## Abstract

**Background:**

The immune checkpoint inhibitor (ICI) anti-PD-L1 monoclonal antibody can inhibit the progress of hepatocellular carcinoma (HCC). Epithelial–mesenchymal transformation (EMT) can promote tumor migration and the formation of immune-suppression microenvironment, which affects the therapeutic effect of ICI. Yin-yang-1 (YY1) is an important transcription factor regulating proliferation, migration and EMT of tumor cells. This work proposed a drug-development strategy that combined the regulation of YY1-mediated tumor progression with ICIs for the treatment of HCC.

**Methods:**

We first studied the proteins that regulated YY1 expression by using pull-down, co-immunoprecipitation, and duo-link assay. The active compound regulating YY1 content was screened by virtual screening and cell-function assay. Isorhamnetin (ISO) and anti-PD-L1 antibody dual-functional mesoporous silica nanoparticles (HMSN-ISO@ProA-PD-L1 Ab) were prepared as an antitumor drug to play a synergistic anti-tumor role.

**Results:**

YY1 can specifically bind with the deubiquitination enzyme USP7. USP7 can prevent YY1 from ubiquitin-dependent degradation and stabilize YY1 expression, which can promote the proliferation, migration and EMT of HCC cells. Isorhamnetin (ISO) were screened out, which can target USP7 and promote YY1 ubiquitin-dependent degradation. The cell experiments revealed that the HMSN-ISO@ProA-PD-L1 Ab nanoparticles can specifically target tumor cells and play a role in the controlled release of ISO. HMSN-ISO@ProA-PD-L1 Ab nanoparticles inhibited the growth of Hepa1-6 transplanted tumors and the effect was better than that of PD-L1 Ab treatment group and ISO treatment group. HMSN-ISO@ProA-PD-L1 Ab nanoparticles also exerted a promising effect on reducing MDSC content in the tumor microenvironment and promoting T-cell infiltration in tumors.

**Conclusions:**

The isorhamnetin and anti-PD-L1 antibody dual-functional nanoparticles can improve tumor immune microenvironment and inhibit YY1-mediated tumor progression. This study demonstrated the possibility of HCC treatment strategies based on inhibiting USP7-mediated YY1 deubiquitination combined with anti-PD-L1 monoclonal Ab.

## Introduction

Hepatocellular carcinoma (HCC) is the most common malignant liver cancer. The prognosis of HCC is poor, and there is a lack of effective drugs for its treatment. HCC progression is also modulated by the immune system. Immune checkpoint inhibitors (ICIs) such as PD-L1 monoclonal antibodies also have a therapeutic effect on HCC [1], but the therapeutic effect needs improvement. Searching for combination drugs of ICIs is important to improve their therapeutic effect on HCC.

Epithelial–mesenchymal transition (EMT) plays a critical role in tumor progression [2]. Tumor cells undergoing EMT can obtain stronger migration and invasion ability. Thus, inhibiting EMT is essential to suppress tumor metastasis. EMT also plays a pivotal role in tumor immunosuppression and immune evasion [3, 4]. The bidirectional regulation between EMT status and PD-L1 expression may also lead to tumor immune escape. Yin-yang-1 (YY1), a transcription factor regulator, is aberrantly expressed in various cancers, where it regulates diverse processes ranging from tumor cell invasion and metastasis to cell survival and proliferation [5–7]. YY1 can regulate EMT directly and/or indirectly by regulating Snail transcription [8]. A number of studies have suggested that YY1 is also closely related to the remodeling of tumor immune microenvironment, and PD-L1 expression is regulated by YY1-mediated signal crosstalk. Such pathways include p53, STAT3, NF-κB, PI3K/AKT/mTOR, and COX-2. YY1 also adjusts the levels of cytokines and growth factors (e.g., IL-6, IL-17, TGF-β, and IFN-γ) [9], thereby modulating the therapeutic effect of tumor immune checkpoint drugs. Inhibiting YY1 expression could have multiple effects on tumor cells, which can not only inhibit the proliferation, migration and EMT of tumor cells, but also enhance the effect of tumor immunotherapy. So inhibiting YY1 expression is a potentially effective strategy for developing combination drugs with immune checkpoint inhibitors (ICIs).

An abnormally high YY1 expression in tumor cells indicates that a stable factor of YY1 exists in tumor cells. Thus, determining the stable factor of YY1 may be an effective way to reduce YY1 levels in tumor cells. In the current work, we first studied the protein that regulated YY1 stability. We found that YY1 can specifically bind to deubiquitinase USP7, which may regulate the malignant progression of HCC by regulating the stability of YY1. We consider that deubiquitination may be one of the important ways to stabilize YY1. Subsequently, virtual screening and cell-function assay were performed to screen the USP7 inhibitors for regulating YY1 expression and inhibiting tumor progression. Isorhamnetin (ISO) can target USP7 and inhibit YY1 expression by inhibiting USP7-mediated YY1 deubiquitination. Given that the effect of tumor immunotherapy can be regulated by YY1, we further proposed a drug-development strategy that combined the regulation of YY1 expression with ICIs.

Mesoporous silica nanoparticles (MSNs) have emerged as promising drug delivery systems due to their unique mesoporous structure, which enables high drug loading capacity and controlled release of drugs [10]. Moreover, the size, shape, and surface chemistry of MSNs can be tailored to specific drug delivery needs, making them versatile carriers for a wide range of drugs. In addition, their good biocompatibility and low toxicity make them attractive candidates for clinical use [11]. Furthermore, MSN production is relatively inexpensive and easy to manufacture, which could lead to future industrial production and widespread clinical application [12]. These advantages indicated the potential of MSNs as a promising platform for drug delivery in cancer therapy.

**Fig. 1:**
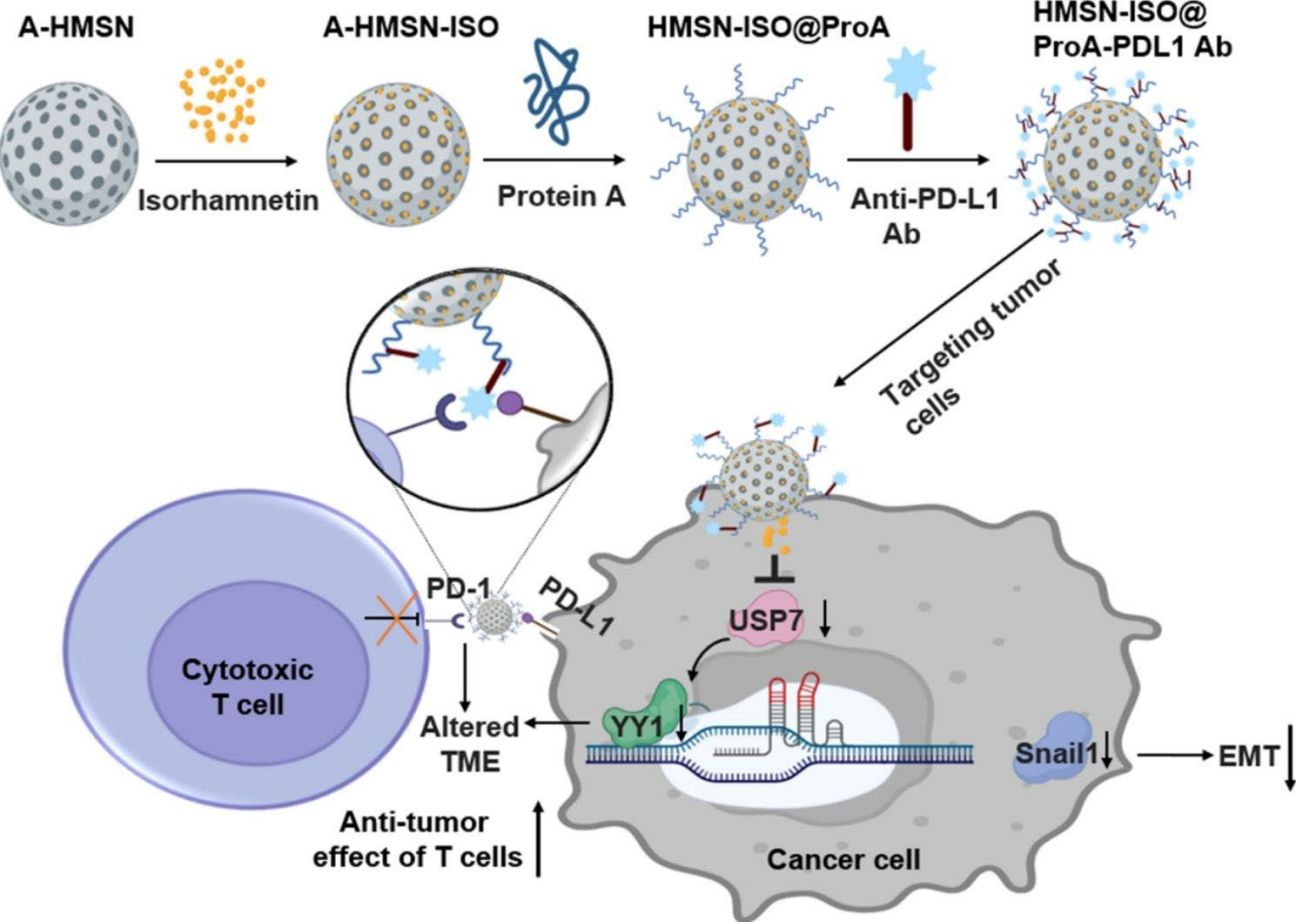
**Schematic of HMSN-ISO@ProA-PD-L1 Ab design route and its antitumor molecular mechanism**

Based on the above background, we designed dual-functional mesoporous silica nanoparticles loading with ISO and anti-PD-L1 monoclonal antibody (HMSN-ISO@ProA-PD-L1 Ab), which can target tumor cells, inhibit YY1 mediated tumor progression, and improve the killing effect of T-cells on tumors by inhibiting the PD-1/PD-L1 signaling pathway (Fig. 1).

## Materials and methods

### Cell culture

All cell lines were purchased from KeyGEN BioTECH (Nanjing, China). The cells were maintained in RPMI 1640 medium with 10% FBS and 1% PS. The cells were stored at 37 °C in an incubator under a humidified atmosphere with 5% CO_2_. The cells were passaged every 3 days.

### Transfection and RNA interference

The recombinant plasmids of Flag–YY1 and Flag–USP7 were transfected using Lipofectamine 2000 (Invitrogen). All siRNAs were transfected using Lipofectamine RNAi MAX following the manufacturer’s recommendations. The final siRNA concentration was 10 nM. Cells were collected after 72 or 96 h.

### Invasion assay

PLC-PRF-5 and HepG2 cells subjected to different treatments were added into the top-chamber inserts coated with Matrigel (BD Biosciences). The bottom chamber was filled with 500 µL of medium containing 10% FBS. The cells were cultured at 37 °C for 24 h. The cells transferred through the filter membrane at the bottom of the chambers were washed three times with 1× PBS, fixed in 4% paraformaldehyde (precooled at 4 °C), and stained with crystal violet staining solution (KeyGEN BioTECH). The invading cells were counted under a microscope (Nikon, Japan).

### Wound-healing assay

PLC-PRF-5, HepG2, and Hepa1-6 cells with different treatments were seeded onto 24-well plates. The cells in the center of the well were scratched. The wound was photographed every 12 h using a light microscope (Nikon, Japan).

### Western blot

The cells were lysed using RIPA lysate, and protein concentrations were detected using a bicinchoninic acid (BCA) assay kit. Then, the proteins were boiled in SDS loading buffer. The proteins from HCC cells were separated by 12% SDS–polyacrylamide gel electrophoresis (PAGE) and transferred onto polyvinylidene difluoride (PVDF) membranes. After blocking the membranes with 5% skimmed milk, they were incubated with primary antibodies, followed by secondary antibodies. The primary antibodies were as follows: anti-USP7 (Abcam, ab108931, 1:1000), anti-YY1 (Affinity, AF3694, 1:1000), anti-E-Cadherin (Abcam, ab1416, 1:1000), anti-vimentin (CST, 5741, 1:1000), and anti-GAPDH (Affinity, AF0911, 1:1000). The proteins on PVDF membranes were detected by enhanced chemiluminescence.

### Pull-down assay

Lysates from PLC-PRF-5 cells expressing Flag–YY1 were prepared using 0.3% Nonidet P-40 lysis buffer (0.2 mM EDTA; 50 mM Tris-HCl, pH 7.4; 150 mM NaCl; 0.3% Nonidet P-40) containing a protease inhibitor cocktail (Roche). Anti-Flag tag (L5) affinity beads (Biolegend) were incubated with the cell extracts for 12 h at 4 °C. After binding with Flag-YY1, the beads were washed with cold 0.1% Nonidet P-40 lysis buffer (0.2 mM EDTA, 50 mM Tris-HCl, 150 mM NaCl, 0.1% Nonidet P-40). Flag peptide (Sigma) was then applied to the beads to elute the Flag protein complex as described by the manufacturer. The eluents were collected and visualized on 10% SDS–PAGE followed by Coomassie Blue Staining. Distinct protein bands were retrieved and analyzed by LC–MS/MS.

### Co-immunoprecipitation (Co-IP) assay

We incubated 50 µL of 50% protein A/G agarose (Pierce) with control or specific antibodies (1–2 µg) for 8 h at 4 °C under constant rotation. PLC-PRF-5 and HepG2 cell lysates were prepared by incubating the cells in 0.3% Nonidet P-40 lysis buffer in the presence of protease inhibitor cocktails. The lysates were centrifuged at 12,000 rpm for 10 min at 4 °C and then incubated with antibody-conjugated beads for an additional 12 h. After incubation, the beads were washed five times using cold 0.1% Nonidet P-40 lysis buffer. The precipitated proteins were eluted from the beads by resuspending the beads in 2× SDS–PAGE loading buffer and boiling for 10 min at 99 °C. The boiled immune complexes were subjected to SDS–PAGE followed by Western blot with USP7 (1:1000) and YY1 (1:1000) antibodies.

### Fast protein liquid chromatography

PLC-PRF-5 cell extracts were separated using Superdex 200 10/300 GL columns (GE Healthcare) and AKTA Explorer Protein Purification System. The column was equilibrated with 1× PBS before use. The column was eluted at a flow rate of 0.5 mL/min, and fractions were collected. Western blot analysis was conducted using USP7 (1:1000) and YY1 (1:1000) antibodies.

### Immunofluorescence assay

The PLC-PRF-5 and HepG2 cells subjected to different treatments were washed three times with 1× PBS, fixed in 4% paraformaldehyde (precooled at 4 °C, Solarbio) for 20 min, and blocked with 5% bovine serum albumin (BSA; KeyGEN BioTECH) containing 0.1% TritonX-100 (Sigma) for 30 min at room temperature. Then, the cells were incubated with anti-E-cadherin (1:50)/anti-vimentin (1:100) or anti-YY1 (1:100)/anti-USP7 antibodies (1:100). The cells were washed again with 1× PBS and incubated with fluorescent conjugated secondary antibodies (1:200, KeyGEN BioTECH) diluted in 5% BSA for about 50 min at room temperature. Finally, the cells were washed with 1× PBS and mounted with DAPI-containing mounting medium (Solarbio). Cell images were taken with a laser scanning confocal microscope.

### Luciferase activity assay

PLC-PRF-5 and HepG2 cells were seeded onto 96-well plates. After 24 h, the plasmids of YY1, USP7, and siRNA were transfected separately into the cells and co-transfected with the luciferase reporter plasmid of the E-cadherin promoter. After 48 h, Gaussia luciferase and secreted alkaline phosphatase (SEAP) luciferase activities were measured consecutively using the Dual-luciferase Reporter Assay System (GeneCopoeia, Inc., USA). Gaussia luciferase was normalized to SEAP activity. The experiment was performed in triplicate.

### Deubiquitination assay

PLC-PRF-5 cells with different treatments were lysed with 0.3% Nonidet P-40 lysis buffer and centrifuged at 12,000 rpm for 10 min. Then, anti-FLAG affinity gel or anti-YY1 antibody-conjugated protein A/G agarose was incubated with the cellular extracts for 12 h at 4 °C. After washing the gels five times with cold 0.1% Nonidet P-40 lysis buffer and boiled in SDS–PAGE loading buffer, they were subjected to SDS–PAGE and Western blot.

### Molecular docking

The crystal structure of USP7 was downloaded from the Protein Data Bank (PDBID: 3IHP). The crystal structure of YY1 was modeled using SWISS-MODEL (https://www.swissmodel.expasy.org/). The protein structure was prepared by adding hydrogen, optimizing the H-bond assignment, assigning bond order, treating disulfides, and performing energy minimization to relax the structure. HEX software was used to perform protein–protein docking. Schrodinger software was used to perform molecule screening. The ligand in the crystal structure was used to define the center site of a docking grid box, and the docking grid box had dimensions of 60 × 60 × 60. The 3D structures of the traditional Chinese medicine molecule were generated with LigPrep and minimized with OPLS-2005 force field. Docking score was used to screen small molecules.

### Lentiviral production and shRNA transfection

The shRNAs targeting USP7 in the pLKO-U6-shRNA, which were carried by pLP1, pLP2, pLP VSV-G assistant vectors, were transfected into HEK293T cells. Viral supernatants were collected 48 h later, clarified by filtration, and concentrated by ultracentrifugation. The PLC-PRF-5 cells were infected with USP7 shRNA plasmids with lentiviruses in vitro to obtain shUSP7 PLC-PRF-5 cells.

### Immunohistochemistry (IHC)

Tissues were deparaffinized with xylene and dehydrated with ethanol. Endogenous peroxidase was blocked by incubating with 3% hydrogen peroxide for 15 min. Antigen retrieval was performed in a steam pressure cooker with citrate-buffered saline (pH 6.0) for 15 min at 95 °C. The tissue section was incubated with normal goat serum for 20 min at room temperature to block unspecific labeling and then incubated with primary antibodies including anti-E-cadherin (1:100,), anti-vimentin (1:100), anti-YY1 (1:100), and anti-USP7 (1:50) antibodies in a humidified chamber overnight at 4 °C. Diaminobenzidine was utilized for color development, and hematoxylin was used as the counterstain. The expression levels of E-cadherin, vimentin, USP7, and YY1 were independently evaluated by two investigators.

### Preparation of the nanodrug HMSN-ISO@ProA-PD-L1 Ab

A-HMSNs (obtained from Xianfeng Nanomaterial Technology Co., Ltd, Jiangsu, China) and ISO (Must Bio-Technology Co.,Ltd, Chengdu, China) were dissolved in absolute ethanol in a 1:1 mass ratio, and thoroughly mixed by ultrasonication for 15 min. After Incubating at room temperature with agitation for 24 h, A-HMSN-ISO was collected by centrifugation at 13,000 rpm for 20 min and washed with ddH_2_O for twice, and all the supernatant and the precipitation was collected separately. The precipitation was further dried using a vacuum dryer. Protein A (1 mg/mL) was activated by EDC/NHS buffer for 60 min and then added into 1 mg/mL A-HMSN-ISO, stirred for 6 h at room temperature, and collected by centrifugation at 13,000 rpm for 20 min, forming HMSN-ISO@ProA. A mixture of 5 µg/mL InVivoMAb anti-mouse PD-L1 (B7-H1) (BioXcell, BE0101) and 1 mg/mL HMSN-ISO@ProA was thoroughly incubated for 3 h with continuous shaking, and the precipitation was collected by centrifugation in the same way to obtain HMSN-ISO@ProA-PD-L1 Ab, which was then dissolved in PBS and stored at 4 °C in darkness.

### Physical characterization of HMSN-ISO@ProA-PD-L1 Ab

DLS and Zeta potential detection were performed at 25 °C using a Zetasizer Nano-ZS (Malvern, Nano-ZS, UK). The particle morphology of HMSN-ISO@ProA-PD-L1 Ab was analyzed through transmission electron microscopy (FEI, Talos L120C G2, Czech). Ultraviolet-visible Spectrophotometer (Evolution 201, USA) was used for ISO drug loading quantification, and the BCA Protein Assay Kit (Thermos, USA) was used for PD-L1 Ab drug loading determination. In order to determine the dissolution rate of ISO in HMSN-ISO@ProA-PD-L1 Ab, we weighed 2 mg of HMSN-ISO@ProA-PD-L1 Ab and prepared a 1 mg/mL sample solution using PBS solution with pH 6.8 and pH 7.5. The sample solution was then placed into a dialysis bag (MWCO 8000 ~ 14000Da) and immersed in 8 mL of the corresponding release solution. The drug release study was conducted at a temperature of 37 °C while protecting the sample from light. At predetermined time points (24 h, 48 h, and 72 h), 2 mL of the release solution was taken out and replaced with fresh PBS solution. The amount of dissolved ISO was then measured using the Ultraviolet-visible Spectrophotometer.

### In vitro toxicity evaluation of HMSN-ISO@ProA-PD-L1 Ab

To detect the effect of drugs on tumor cell viability, Hepa1-6 cells were treated with different drugs. Except for the A-HMSN group, the dosage of other groups was quantified based on ISO to ensure that the ISO content was 30 µM. After 24 and 48 h of incubation, the culture medium was aspirated, the cells were washed with PBS and subsequently added with fresh complete medium containing serum and 10 µL of CCK8 (Cell Counting Kit-8, Solarbio, China), and incubated at 37 °C for 2 h. Absorbance at 450 nm was measured using a microplate reader (TECAN, Spark, Switzerland).

### Carboxyfluorescein succinimidyl ester (CFSE) assay

To evaluate the effect of drugs on cell proliferation in vitro CFSE dilution assay was performed. As directed by a CFDA SE Cell Proliferation Assay and Tracking Kit (Beyotime, China), CFSE dye was added to Hepa1-6 and T-cells in good condition for adequate labeling in darkness at 37 ℃ for 15 min. The labeled cells were then incubated at 37 °C at 5% CO_2_ and 98% O_2_ for 48 h. After harvesting the cells, the proliferation of CFSE-labeled cells was determined by flow cytometry.

### Targeting effect analysis of HMSN-ISO@ProA-PD-L1 Ab

Cell suspensions of Hepa1-6 cells and Raw264.7 cells were prepared in the same way as for cell passage. DiI dye (2 µL) was added to the Hepa1-6 cells suspension, 10 µL of Hoechst33342 dye was added to the Raw264.7 cell suspension, and they were mixed thoroughly. After incubating for 20 min and centrifuged at 800 rpm/min for 5 min to remove the staining solution, PBS was used for washing three times. After thorough mixing in 1:1 ratio, the cells were added to a DMEM dish containing 10% serum and incubated in a cell constant-temperature incubator. The cells were treated with fluorescently labeled HMSN-ISO@ProA-PD-L1 Ab for 6 h, followed by photographing with fluorescence imaging microscopy (total internal reflection fluorescent microscope, TIRF & Thunder, DMi8S, Germany) to observe the uptake of the cells.

### PLC-PRF-5 tumor xenograft model

The PLC-PRF-5 cells and shUSP7 PLC-PRF-5 cells (2 × 10^6^) in PBS were injected into BALB/c nude mice (6–8 weeks old; Charles River, Beijing, China) by subcutaneous injection. Tumors were measured every 3 days using a vernier caliper, and the volume was determined using the formula *V* = *ab*^2^/2 (*a* = tumor length, *b* = tumor width). Afterwards, the mice were euthanized, and the tumor and lung tissues were harvested, fixed in 4% paraformaldehyde, and embedded in paraffin for histologic examination or hematoxylin and eosin (H&E) staining.

### Antitumor efficiency evaluation of HMSN-ISO@ProA-PD-L1 Ab in vivo

Seven-week-old female C57BL/6 mice (Vital River, China) were acclimated under standard laboratory conditions (ventilated room, 25 ± 1 °C, 60% ± 5% humidity, 12 h light/dark cycle) and had free access to standard water and food. To observe the antitumor effect of HMSN-ISO@ProA-PD-L1 Ab, Hepa1-6 cells (2 × 10^6^ cells) were injected subcutaneously into the right hind leg of the mice. When the tumor volume approximately reached about 100 mm^3^ (9 days after injection), different drugs were intratumorally injected into the mice every three days for four times according to the following grouping (n = 6): (i) Control group (administration with equal volume of PBS), (ii) ISO (125 µg/mice), (iii) anti-PD-L1 antibody (1.52 µg/mice), and (iv) HMSN-ISO@ProA-PD-L1 Ab (308 µg/mice, the ISO content in the nanoparticles were equal to the ISO administration group). Tumors were measured every 3 days using a vernier caliper. Afterwards, the mice were euthanized on day 24, and the tumor and major organs (i.e., kidneys, spleens, livers, and lungs) were harvested, fixed in 4% paraformaldehyde, and embedded in paraffin. Tissues embedded in paraffin were sliced into 5 μm thick sections, dried, and set aside for the subsequent immunofluorescent, immunohistochemical, H&E, and TUNEL staining (TUNEL apoptosis detection kit (FITC), Yeasen, China).

### Flow Cytometry Analysis of myeloid-derived suppressor cells (MDSCs) and T-cells content

The tumors were harvested and digested with collagenase IV. The resulting cells were lysed with red blood cell lysis buffer (Beyotime, China) and passed through nylon mesh filters (70 μm). To analyze the T-cells, single-cell suspensions were resuspended in PBS labeling solution with CD45 (30-F11, Invitrogen), CD3 (17A2, BD), and CD8 (53–6.7, BD). CD11b (M1/70, Invitrogen) and Gr-1 (RB6–8C5, Invitrogen) were labeled single cells for MDSCs.

### Statistical analysis

Data are presented as the mean ± standard deviation (SD) with error bar. Two-tailed unpaired Student’s t-test was used to compare two groups of data. One-way ANOVA was used to compare multiple groups of data. P < 0.05 was considered statistically significant.

## Results

### YY1 have physical interaction with USP7

**Fig. 2:**
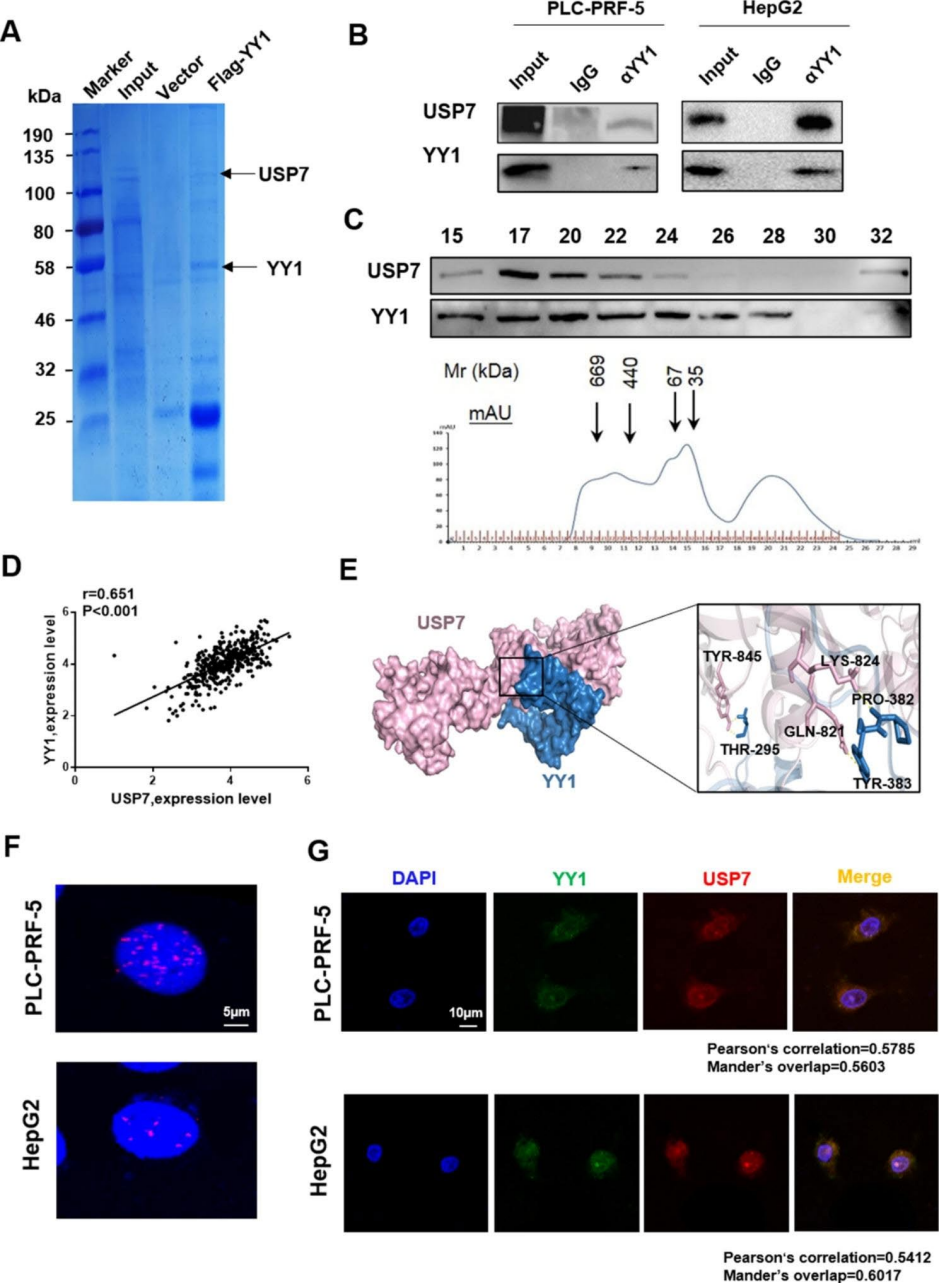
**YY1 and USP7 have a physical interaction.** **(A)** Interaction protein of YY1 in PLC-PRF-5 cells as detected by Flag pull-down affinity-purification experiment. The protein samples were stained with Coomassie Blue, and the proteins interacting with YY1 were detected by MS. **(B)** USP7 and YY1 interaction detected by Co-IP. **(C)** Western blot analysis of the USP7 and YY1 contents in each component of the whole-cell extracts of PLC-PRF-5 separated by FPLC. **(D)** USP7 and YY1 expression levels in HCC were positively correlated. Data were obtained from TCGA (P < 0.001, r = 0.651). **(E)** Diagrams of the molecular-docking simulation of YY1 (right) and USP7 (left) and the interacting amino acid sites of YY1 and USP7. **(F)** YY1 and USP7 interaction in PLC-PRF-5 and HepG2 cells as detected by duo-link PLA. **(G)** USP7 and YY1 interaction in PLC-PRF-5 and HepG2 cells as detected by immunofluorescence assay

In order to study the protein that regulated YY1 stability, the whole-cell extracts of PLC-PRF-5 cells with or without Flag–YY1 overexpression were subjected to affinity purification using anti-Flag affinity gel and mass spectrometry (MS) analysis to identify the proteins that interact with YY1 (Fig. 2A). USP7 was identified in the Flag-YY1 protein complex. Co-immunoprecipitation (Co-IP) experiment was performed to verify the interaction between YY1 and USP7. Results showed that YY1 could be co-immunoprecipitated with USP7 in PLC-PRF-5 cell extracts. Similar results were found in HepG2 cells (Fig. 2B). Fast protein liquid chromatography (FPLC) experiment was performed to further confirm the interaction between YY1 and USP7. Results also showed that YY1 and USP7 in PLC-PRF-5 cells coexisted in the multiprotein complex. The overlap of YY1 and USP7 peaked at fraction 17 (Fig. 2C). Results of the expression correlation analysis of USP7 and YY1 obtained from The Gene Expression Profiling Interactive Analysis database (http://gepia.cancer-pku.cn) showed that the expression levels of USP7 and YY1 were positively correlated (P < 0.001, r = 0.625; Fig. 2D). The protein–protein interaction interface was evaluated through the computer simulation of USP7 and YY1. At the interaction interface, three pairs of amino acids interacted with each other (TYR-845/THR-295, GLN-821/TYR-383, and LYS-824/PRO-382; Fig. 2E). The interaction between YY1 and USP7 was observed by proximity ligation assay (PLA). Results showed that YY1 interacted with USP7 (the red dot indicates protein interaction) in PLC-PRF-5 and HepG2 cells (Fig. 2F). Double-labeled immunofluorescence staining with YY1 and USP7 antibodies was performed on PLC-PRF-5 and HepG2 cells to further verify the interaction between USP7 and YY1. The experimental results showed that YY1 and USP7 had obvious co-localization (Fig. 2G).

### USP7 stabilized YY1 expression in HCC cells through its deubiquitination activity

**Fig. 3:**
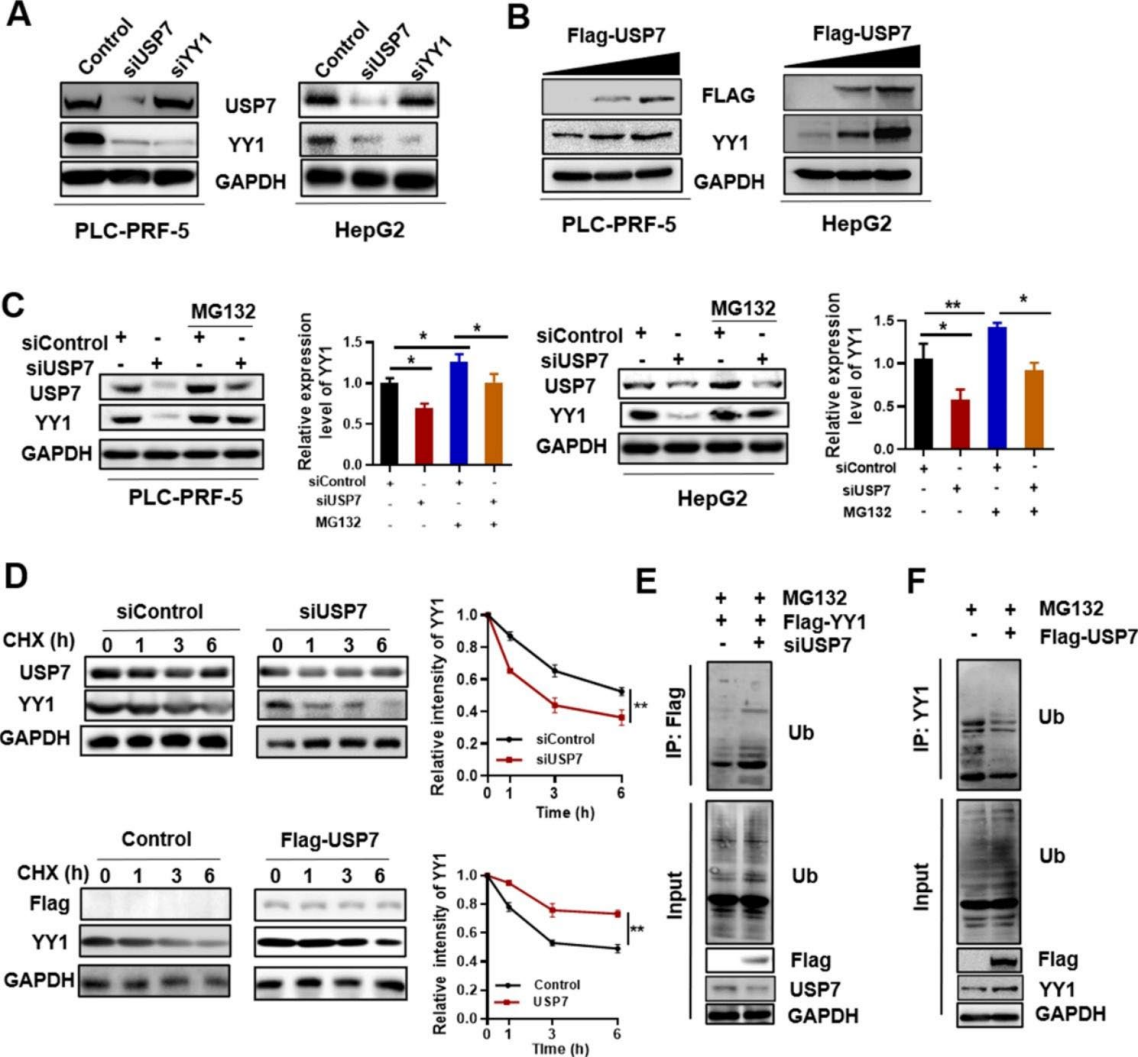
**USP7 stabilizes YY1 expression through deubiquitination.** **(A)** Effect of USP7 on YY1 expression in PLC-PRF-5 and HepG2 cells as detected by Western blot. **(B)** Changes in YY1 expression after transfection with different amounts of Flag-USP7 vector. **(C)** Western blot analysis of the change in YY1 expression in siUSP7 PLC-PRF-5 and HepG2 cells treated with or without MG132. **(D)** Effect of USP7 on YY1 degradation in PLC-PRF-5 cells transfected with siUSP7 or Flag-USP7 vector and treated with CHX. **(E)** Effect of USP7 on YY1 ubiquitination in PLC-PRF-5 cells treated with siUSP7 and Flag-YY1. Flag beads were used for immunoprecipitation. **(F)** Effect of USP7 on YY1 ubiquitination in PLC-PRF-5 cells treated with Flag-USP7. YY1 antibody was used for immunoprecipitation. Data are expressed as the mean ± SD (*P < 0.05, **P < 0.01)

SiUSP7 or siYY1 RNA was transfected into PLC-PRF-5 and HepG2 cells to verify the interaction between USP7 and YY1 and the functional importance of their spatial co-localization. Interfering with USP7 can reduce YY1 content in cells, whereas interfering with YY1 had no influence on USP7 content (Fig. 3A). USP7 overexpression increased YY1 content in PLC-PRF-5 and HepG2 cells in a dose-dependent manner (Fig. 3B). Proteasome inhibitor MG132 can also reverse the decrease in YY1 protein induced by siUSP7 (Fig. 3C). Therefore, we suspected that USP7 can stabilize YY1 protein by inhibiting the ubiquitin-mediated proteasome degradation of YY1. The protein synthesis inhibitor, cycloheximide (CHX), was used to detect the half-life of YY1 in USP7 knockdown PLC-PRF-5 cells to confirm this finding (Fig. 3D). Results showed that the half-life of YY1 was shortened in the siUSP7-treated group and prolonged in the USP7-overexpression group. The above results showed that USP7 may regulate the stability of YY1 by inhibiting YY1 ubiquitination. Considering the role of USP7, we speculated that USP7 may affect the stability of YY1 through its activity of deubiquitination. Ubiquitination-detection results showed that YY1 ubiquitination increased in USP7-interfered PLC-PRF-5 cells and decreased in USP7-overexpressed PLC-PRF-5 cells (Fig. 3E and F). These results indicated that USP7 stabilized YY1 expression by deubiquitinating YY1.

### USP7 promoted migration, invasion, and EMT of HCC cells

**Fig. 4:**
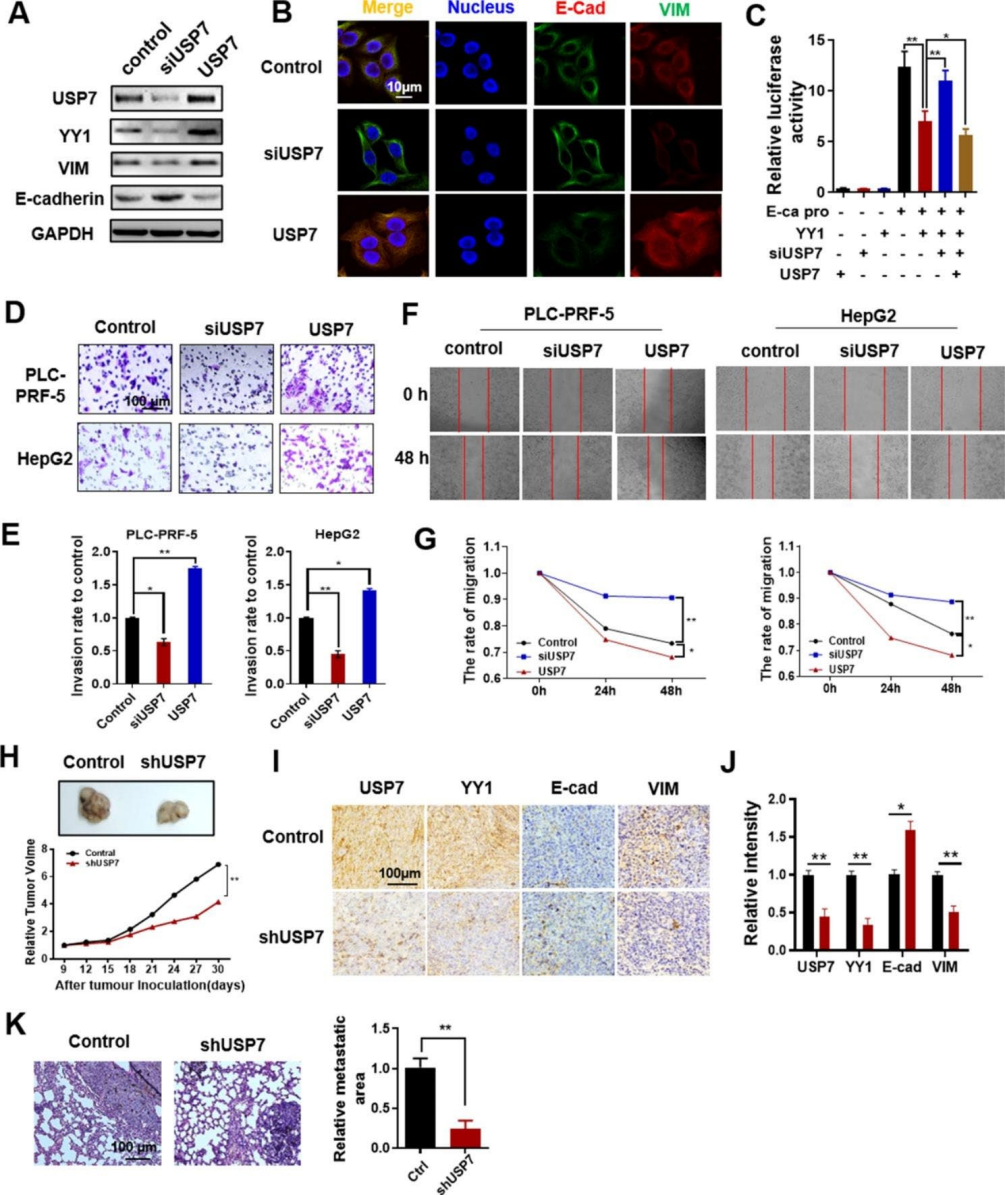
**USP7 promotes the EMT of PLC-PRF-5 and HepG2 cells.** **(A)** Western blot analysis of E-cadherin and vimentin in PLC-PRF-5 cells treated with USP7 siRNA or USP7 overexpression vector. **(B)** Immunofluorescence analysis of EMT markers E-cadherin and vimentin in PLC-PRF-5 cells treated with USP7 siRNA or USP7 overexpression vector. **(C)** Effect of USP7 on E-cadherin transcription level in PLC-PRF-5 cells transfected with E-cadherin-dependent reporter plasmid. **(D, E)** Effect of USP7 on the invasion ability of HCC cells. **(F, G)** Effect of USP7 on the migration ability of HCC cells. **(H)** Tumor tissue images and tumor volume statistical curves of shUSP7 PLC-PRF-5 tumor-bearing mice. **(I, J)** IHC staining results and staining intensity statistics of USP7, YY1, E-cadherin, and vimentin in tumor tissue sections of each group. **(K)** Lung tissue sections (H&E staining) and statistical data of relative lung metastasis area in each group. Data are expressed as the mean ± SD (*P < 0.05, **P < 0.01)

YY1 was a key regulation protein of EMT. The effect of USP7 on EMT was studied to understand the biological function of USP7-mediated YY1 stabilization. Western blot analysis showed that E-cadherin expression increased and vimentin expression decreased in USP7-knockdown PLC-PRF-5 cells (Fig. 4A). In the USP7-overexpression cells, E-cadherin expression decreased and vimentin expression increased. E-cadherin and vimentin expression levels were also detected by immunofluorescence (Fig. 4B). Results showed the same trend as the Western blot findings. Luciferase reporter gene analysis further showed that USP7 knockdown decreased the transcription-inhibition ability of YY1 on E-cadherin, whereas USP7 overexpression promoted the transcription-inhibition ability of YY1 on E-cadherin (Fig. 4C). Transwell and wound-healing experiments showed that USP7 knockdown inhibited the invasion (Fig. 4D and E) and migration (Fig. 4F and G) activity of HCC cells, and USP7 overexpression promoted the invasion and migration ability of HCC cells.

USP7 knockdown PLC-PRF-5 cells (using shUSP7 vector) were transplanted into BALB/c nude mice to establish xenograft tumor models and investigate the effect of USP7 on HCC progression in vivo. Results showed that USP7 knockdown can slow the growth rate of PLC-PRF-5 xenografts (Fig. 4H). IHC staining was performed to detect the expression levels of USP7, YY1, E-cadherin, and vimentin in tumor tissues. USP7, YY1, and vimentin expression decreased, whereas E-cadherin expression increased in the shUSP7 group (Fig. 4I and J). Lung metastases also decreased in USP7-deficient xenografts (Fig. 4K). These results suggested that interference with USP7 can reduce YY1 expression and thus inhibited the migration, invasion, and EMT of HCC cells.

## USP7 and YY1 influenced HCC prognosis

**Fig. 5:**
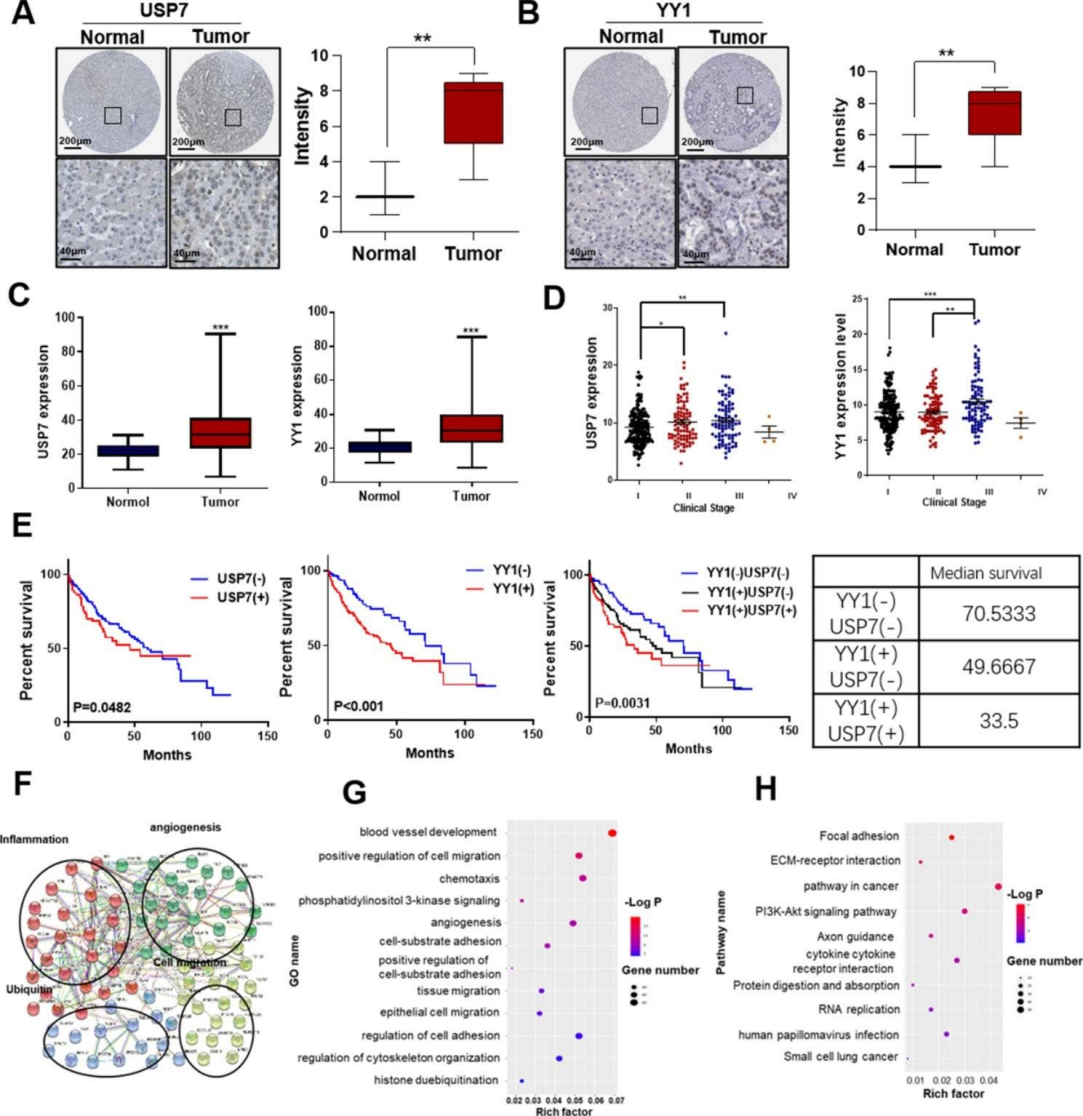
**Effects of USP7 and YY1 on liver cancer prognosis.** **(A, B)** IHC analysis of USP7 and YY1 protein expression levels in tumors and normal liver tissues. Data were obtained from TCGA. **(C)** USP7 and YY1 expression levels in primary tumors (n = 371) and normal liver tissues (n = 50). **(D)** Statistical diagram of the relationship between USP7/YY1 expression and clinical stage of liver cancer. **(E)** Survival analysis of USP7 and YY1 in liver cancer clinical data from TCGA. Kaplan–Meier curve showing the 5-year survival rate of TCGA-LIHC samples classified by USP7 or YY1 expression (n = 346). **(F-H)** Protein–protein interaction (F), GO (G), and KEGG (H) analysis of the differential gene enrichment of tumor tissues in the USP7 high-expression group. Data were obtained from TCGA.

Clinical data were analyzed to determine the effect of USP7 and YY1 on liver cancer. The clinical data of liver cancer were obtained from The Cancer Genome Atlas (TCGA) database. IHC results showed that USP7 and YY1 expression levels were higher in tumor tissues than in normal liver tissues (Fig. 5A and B). From the University of Alabama Cancer database (http://ualcan.path.uab.edu/index.html), the transcriptional analysis of TCGA samples also showed that USP7 and YY1 were highly expressed in liver cancer tissues compared with normal liver tissues (Fig. 5C). Moreover, USP7 and YY1 expression levels were positively correlated with the clinical stage of liver cancer in TCGA (Fig. 5D). Survival analysis also showed that the group with high expression of USP7 and YY1 showed the worst prognosis of HCC (Fig. 5E). Gene analysis also showed that the function and signal pathway of differential gene enrichment in the USP7 high-expression group were related to cell migration, chemotaxis, cell adhesion, angiogenesis, deubiquitination, and the PI3K-Akt pathway (Fig. 5F-H). The above results showed that USP7-YY1 interaction was involved in the malignant progression of HCC, supporting the possibility that targeting USP7-mediated YY1 ubiquitination may be a strategy for HCC treatment.

### ISO targeted USP7 and promoted ubiquitination-depended degradation of YY1

**Fig. 6:**
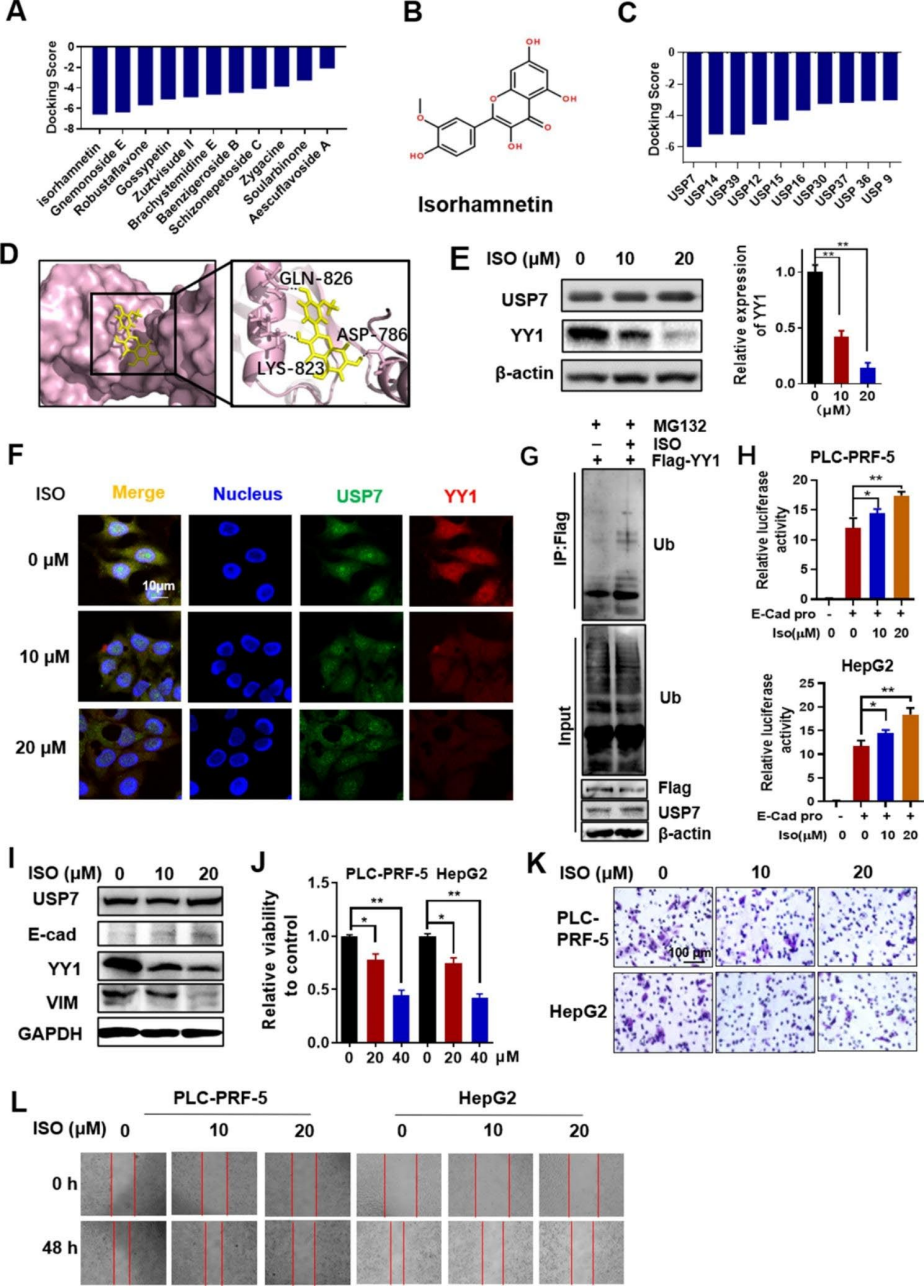
**Isorhamnetin (ISO) inhibits USP7 deubiquitination activity and promotes YY1 ubiquitination degradation.** **(A)** Molecular-docking screening results of traditional Chinese medicine monomers interacting with USP7. **(B)** The chemical structure of ISO. **(C)** Molecular-docking results of ISO with members of the USP family. **(D)** Molecular-docking simulation of ISO binding to USP7. **(E)** Western blot analysis of YY1 expression in ISO-treated PLC-PRF-5 cells. **(F)** Expression of USP7 and YY1 in PLC-PRF-5 cells treated with different concentrations of ISO as detected by immunofluorescence. **(G)** Effect of ISO on YY1 ubiquitination. PLC-PRF-5 cells were treated with Flag-YY1 and MG132. Flag beads were used for immunoprecipitation. **(H)** Luciferase activities of PLC-PRF-5 and HepG2 cells treated with ISO as determined by the transfection of E-cadherin-dependent reporter plasmid. **(I)** Expression of EMT markers in PLC-PRF-5 cells treated with ISO. **(J)** Effect of ISO on the vitality of PLC-PRF-5 and HepG2 cells. **(K)** Effects of ISO on the invasion of PLC-PRF-5 and HepG2 cells. **(L)** Effects of ISO on the migration of PLC-PRF-5 and HepG2 cells. Data are expressed as the mean ± SD (*P < 0.05, **P < 0.01)

Based on the above results, we aimed to screen active molecules that inhibited the deubiquitination effect of USP7 on YY1, which can further inhibit the effect of YY1 on EMT and inhibit the malignant progression of HCC. According to the structure of USP7, the leading compound with the highest docking score with USP7 was selected from the database of traditional Chinese medicine through virtual screening. Results showed that ISO had the best binding ability with USP7 (Fig. 6A and B). Molecular-docking results also showed that ISO had the highest docking score with USP7 among the USP family members. This finding revealed the specificity of ISO’s binding to USP7 (Fig. 6C). The binding sites of ISO and USP7 were shown in Fig. 6D. PLC-PRF-5 cells were treated with different concentrations of ISO to explore the effect of ISO on USP7 and YY1. Western blot analysis showed that YY1 expression decreased and USP7 expression did not change substantially in ISO-treated cells (Fig. 6E). According to immunofluorescence assay results, ISO did not affect USP7 expression but decreased YY1 expression dose dependently (Fig. 6F). Subsequently, the ubiquitin level of YY1 was detected in ISO (20 µM)-treated PLC-PRF-5 cells (Fig. 6G). YY1 ubiquitin level increased in the ISO-treated groups. These results showed that ISO targeted USP7 and inhibited the deubiquitination effect of USP7 on YY1.

Luciferase reporter gene analysis was performed to investigate the inhibitory effect of ISO on EMT. PLC-PRF-5 and HepG2 were transfected with E-cadherin-dependent reporter plasmid to determine the effect of ISO on E-cadherin. Results showed that ISO increased the transcription activity of E-cadherin in a dose-dependent manner (Fig. 6H). Western blot analysis further showed that ISO treatment in PLC-PRF-5 cells increased E-cadherin expression and decreased vimentin expression (Fig. 6I). Moreover, cell-viability experiment (Fig. 6J), Transwell (Fig. 6K), and wound-healing experiment (Fig. 6L) in PLC-PRF-5 and HepG2 cells showed that ISO inhibited HCC cells’ viability, invasion and migration abilities.

### HMSN-ISO@ProA-PD-L1 Ab can specifically target tumor cells to perform antitumor function

**Fig. 7:**
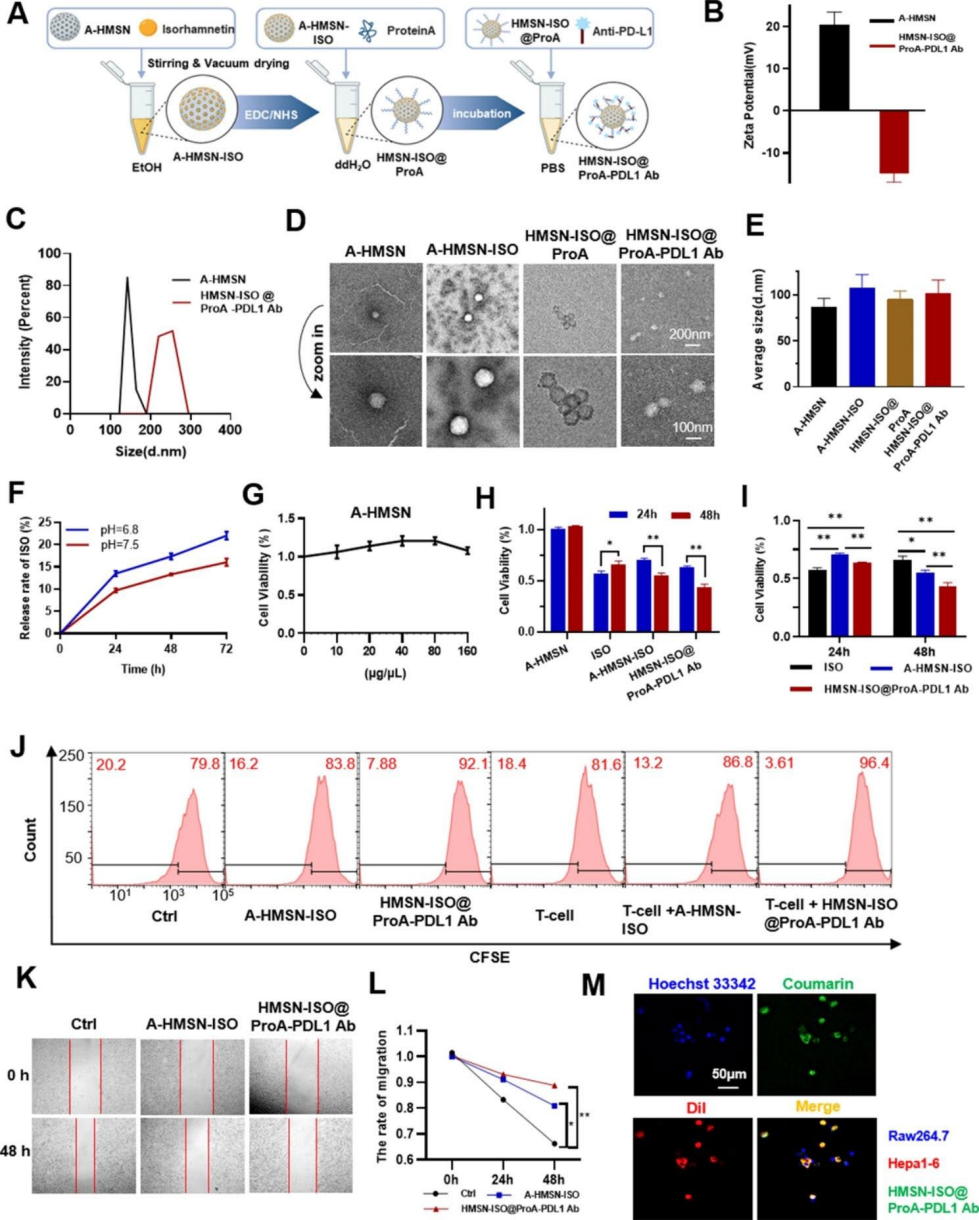
**HMSN-ISO@ProA-PD-L1 Ab can specifically target tumor cells to perform antitumor function.** **(A)** Schematic of the preparation of HMSN-ISO@ProA-PD-L1 Ab. **(B, C)** Zeta Potential and particle-size distribution of A-HMSN and HMSN-ISO@ProA-PD-L1 Ab as determined by dynamic light scattering. **(D)** Cryo-TEM image of A-HMSN, A-HMSN-ISO, HMSN-ISO@ProA and HMSN-ISO@ProA-PD-L1 Ab. **(E)** The statistical results of the average diameter of the particles under TEM. **(F)** The release rate of ISO in HMSN-ISO@ProA-PD-L1 Ab nanomaterials in PBS of pH 6.8 and pH 7.5 at 24 h, 48 h, and 72 h. **(G)** Effect of different concentrations of A-HMSN on cell viability at 48 h detected by CCK-8 assay. **(H, I)** Effects of ISO (30 µM), A-HMSN-ISO, and HMSN-ISO@ProA-PD-L1 Ab on cell viability at 24 and 48 h detected by CCK-8 assay. The dosage of A-HMSN-ISO and HMSN-ISO@ProA-PD-L1 Ab was adjusted to achieve ISO content of 30 µM. **(J)** CFSE histograms of Hepa1-6 cells treated with HMSN-ISO and HMSN-ISO@ProA-PD-L1 Ab followed by continued incubation for 48 h after T-cell exposure or in the absence of T-cells. The dosage was adjusted to ISO content of 10 µM. **(K, L)** Effects of A-HMSN-ISO and HMSN-ISO@ProA-PD-L1 Ab on cell migration detected by wound-healing assay. The dosage was adjusted to ISO content of 10 µM. **(M)** Fluorescence images of coumarin-labeled HMSN-ISO@ProA-PD-L1 Ab (adjusting to ISO content of 10 µM) selectively targeting Hepa1-6 cells under co-culture conditions with Raw264.7 cells in a 1:1 ratio. All values are expressed as the mean ± SD, ns, not significant, *P < 0.05, **P < 0.01

The effect of tumor immunotherapy can be regulated by YY1, so we proposed a drug-development strategy that combined the regulation of YY1 expression with anti-PD-L1 monoclonal Ab. The inhibitory effect of ISO on YY1-mediated EMT combined with anti-PD-L1 antibody may reprogram the tumor immune microenvironment and enhance the antitumor effect of tumor immunocheckpoint inhibitors. Amine-functionalized HMSNs (A-HMSNs) with good biocompatibility, ordered mesoporous structure, large specific surface area, and easily decorated surface are extensively used [13, 14], which were applied to the carrier of ISO and anti-PD-L1 monoclonal antibody (PD-L1 Ab). We first used A-HMSN to adsorb ISO (A-HMSN-ISO) and then covalently modified the surface of A-HMSN with Protein A (HMSN-ISO@ProA), which can further bind with PD-L1 Ab. On this basis, HMSN-ISO@ProA-PD-L1 Ab loaded with ISO and anti-PD-L1 monoclonal antibody were prepared (Fig. 7A). After loading A-HMSN with ISO and anti-PD-L1 Ab, the median zeta potential changed from + 20.36 mV to -14.92 mV (Fig. 7B). The particle size determined by DLS increased from 153 ± 11 nm to 237 ± 17 nm (Fig. 7C). The morphology of the nanoparticles was detected using cryo-transmission electron microscopy (TEM) (Fig. 7D). We also measured the average diameter of the particles under TEM using ImageJ software. The results showed that the average particle size of HMSN-ISO@ProA-PD-L1 Ab measured by TEM was 101.7 ± 8.2 nm (Fig. 7E). Due to preparation in ethanol, A-HMSN-ISO had larger particle size than the A-HMSN group, and similar phenomena have also been reported in literature [15]. The statistical results also showed that the nanoparticle size measured by DLS is larger than that measured by TEM. The main reason for the difference in particle size between DLS and electron microscopy is that DLS measures the size of composite particles, which are hydrated particles in solution, while electron microscopy measures the size of dry particles.

The drug loading tests of ISO and PD- L1 Ab in HMSN-ISO@ProA-PD-L1 Ab were also detected. The ISO drug loading was 0.406 mg/mg in the nanoparticles. The PD-L1 Ab had a drug load of 4.931 µg/mg detected by BCA Protein Assay Kit. To determine the dissolution rate of ISO in HMSN-ISO@ProA-PD-L1 Ab, we measured the concentration of ISO at different time points in separate PBS solutions with pH 6.8 and pH 7.5. The results showed that ISO was slowly released from the nanomaterials over time in a time-dependent manner (Fig. 7F). The release rate of ISO in the pH 6.8 solution was higher than that in the pH 7.5 solution, indicating that it has a better release rate in the tumor microenvironment.

Then the in vitro biological activity studies were conducted. Firstly, the effect of A-HMSN (blank nanoparticle control) on cell viability were detected. A-HMSN showed no significantly cytotoxicity to Hepa1-6 tumor cells, indicating that A-HMSN mainly acted as a drug carrier (Fig. 7G). Then Hepa1-6 cells were treated with A-HMSN, ISO, A-HMSN-ISO, and HMSN-ISO@ProA-PD-L1 Ab for 24 and 48 h, respectively. The results showed that ISO had a good inhibitory effect on tumor cell viability at 24 h, but its effect was weakened at 48 h. The inhibitory effect of A-HMSN-ISO and HMSN-ISO@ProA-PDL1 Ab at 48 h was significantly improved compared to 24 h, and both were more effective than the ISO group at 48 h. Moreover, HMSN-ISO@ProA-PDL1 Ab exhibited the strongest inhibitory ability at 48 h, possibly due to the targeted binding effect of PD-L1 Ab in nanoparticles with PD-L1 receptors of tumor cells (Fig. 7H and I). We further tested whether HMSN-ISO@ProA-PDL1 Ab can enhance the cytotoxic effect of T-cells on tumor cells. T-cells derived from the spleen of wild-type C57BL/6 mice were co-cultured with Hepa1-6 cells (2:100), and the proliferation capacity of HMSN-ISO@ProA-PD-L1 Ab treated co-cultured tumor cells was significantly weaker than the co-cultured tumor cells of the control group (Fig. 7J). The possible reason was that anti-PD-L1 Ab acted as an immune checkpoint blocker, which increased the lethality of T-cells. The migration ability of tumor cells after HMSN-ISO@ProA-PD-L1 Ab treatment was significantly reduced (Fig. 7K and L). To study the targeting effect of HMSN-ISO@ProA-PD-L1 Ab, Hepa1-6 and Raw264.7 cells were co-cultured and stained with different fluorescent dyes. Hepa1-6 cells membrane was marked red with DiI, Raw264.7 nucleus was stained blue with Hoechst 33342, and HMSN-ISO@ProA-PD-L1 Ab was marked as green fluorescence with coumarin. Fluorescence assay showed that the green fluorescence primarily appeared in Hepa1-6 cells (Fig. 7M), indicating that HMSN-ISO@ProA-PD-L1 Ab could specifically target tumor cells and could escape the phagocytosis of macrophages to a certain extent. Combined with the above characterization results, the nanoparticles loaded with ISO and anti-PD-L1 Ab had more significant effects on inhibiting the proliferation and migration of tumor cells than the same amount of ISO, thereby achieving good targeting and sustained-release effects.

#### HMSN-ISO@ProA-PD-L1 Ab can inhibit the progression of tumors in Hepa1-6 tumor-bearing model

**Fig. 8:**
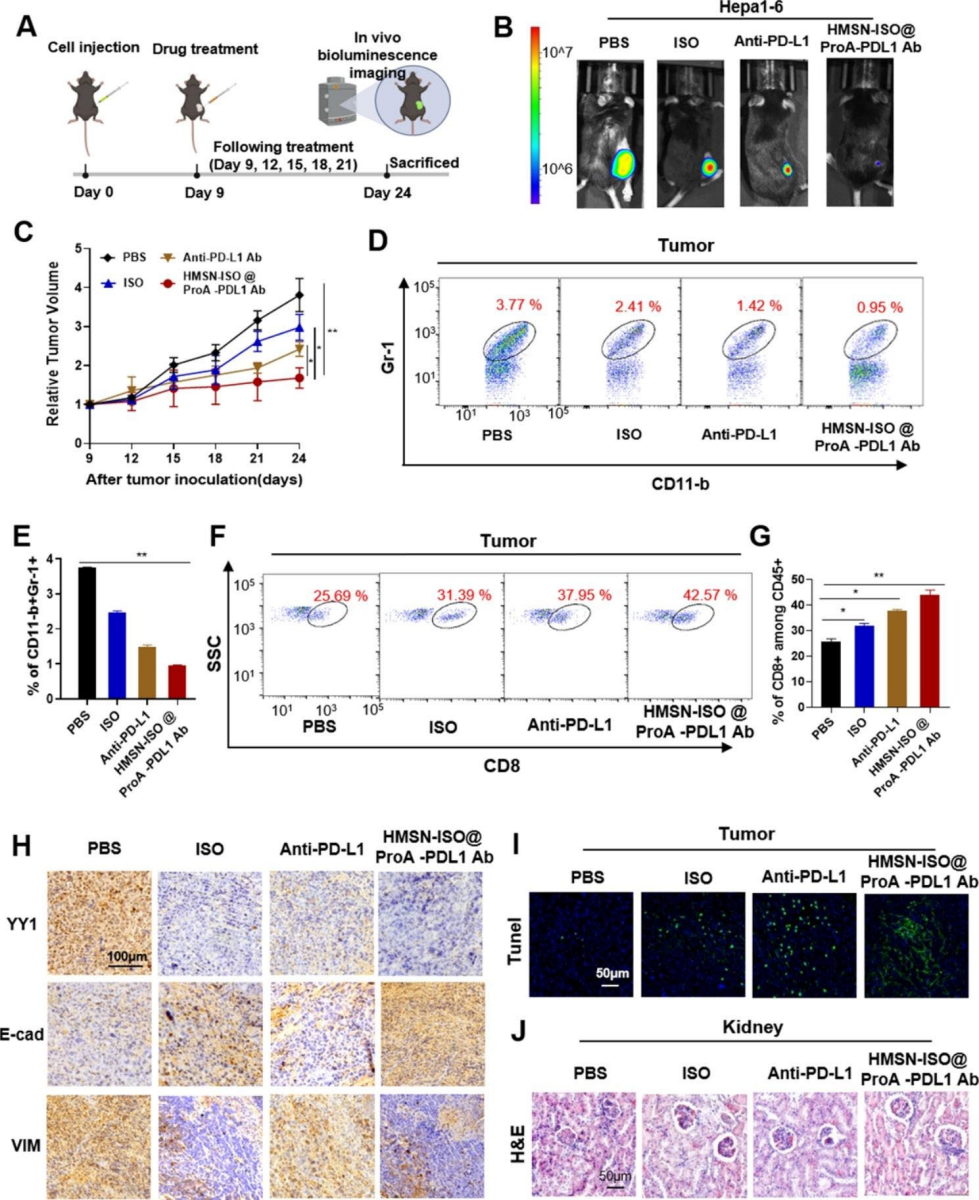
**HMSN-ISO@ProA-PD-L1 Ab inhibits tumor progression in Hepa1-6 transplanted mice.** **(A)** Schematic of the experimental setup to test the effects of ISO, anti-PD-L1 Ab, and HMSN-ISO@ProA-PD-L1 Ab on Hepa1-6 subcutaneously transplanted C57BL/6 mice. **(B)** Bioluminescence images of Hepa1-6-Luc tumor transplanted mice at the end of the experiment. **(C)** Growth curve of Hepa1-6 tumors implanted in C57BL/6 mice after treatment with HMSN-ISO@ProA-PD-L1 Ab. **(D, E)** Flow cytometry analysis of the proportion of myeloid-derived suppressor cells (MDSCs) in tumor tissues after drug treatment. **(F, G)** Flow cytometry analysis of the proportion of CD8 + T-cells in tumor tissues. **(H)** IHC analysis of changes in YY1, vimentin, and E-cadherin levels in tumor tissues after drug treatment. **(I)** Effects of HMSN-ISO@ProA-PD-L1 Ab on the apoptosis of tumor tissues in mice detected by TUNEL staining. **(J)** Representative H&E-stained kidney sections after drug treatment. All values are expressed as the mean ± SD, *P < 0.05, **P < 0.01

To study the effect of HMSN-ISO@ProA-PD-L1 Ab in vivo, Hepa1-6 cells (2 × 10^6^) were injected under the skin of the right hind leg of wild-type C57BL/6 mice, and drug treatment was started when the tumor grew to 50–100 mm^3^ (Fig. 8A). The tumor growth rate of mice during treatment was PBS control group > ISO group > PD-L1 group > HMSN-ISO@ProA-PD-L1 Ab group, indicating that HMSN-ISO@ProA-PD-L1 Ab can effectively inhibited the growth of Hepa1-6 tumors (Fig. 8B and C). Flow cytometry was used to analyze the proportion of myeloid-derived suppressor cells (MDSCs) and T-cells in Hepa1-6 tumors. Results showed that the tumor tissues of mice treated with HMSN-ISO@ProA-PD-L1 Ab contained less MDSC (Fig. 8D and E) and more CD8^+^ T-cell numbers (Fig. 8F and G) than other groups. Results showed that ISO may also change the tumor immune microenvironment by reducing the level of YY1 and enhance the immune killing ability of T-cells. IHC staining verified a significant decrease in YY1, a significant decrease in vimentin expression and a significant increase in E-cadherin expression in tumor tissues of the HMSN-ISO@ProA-PD-L1 Ab treated group (Fig. 8H), indicating that HMSN-ISO@ProA-PD-L1 Ab played a role in inhibiting EMT of tumors. We also investigated the degree of apoptosis in tumor tissues through TUNEL staining. Results showed that the number of apoptotic cells in tumor tissues of HMSN-ISO@ProA-PD-L1 Ab treated mice significantly increased compared with those in other groups (Fig. 8I). The safety of HMSN-ISO@ProA-PD-L1 Ab in vivo was also studied, and HE staining showed that HMSN-ISO@ProA-PD-L1 Ab treated mice kidney had no obvious lesions (Fig. 8J).

## Discussion

HCC was a common malignant tumor of the digestive system [16, 17]. Its mortality rate ranks the second among cancers. Early HCC diagnosis was difficult, and the recurrence rate was high, making the prognosis poor. HCC treatment includes interventional ablation, transcatheter arterial chemoembolization, surgical resection, and chemotherapy [18]. Recent studies have shown that ICIs also have a certain therapeutic effect on HCC. This finding has led to the accelerated FDA approval of nivolumab and pembrolizumab in HCC. However, clinical results are not as expected. We speculated that therapy failure may be attributed to a strongly immunosuppressive tumor microenvironment in HCC [19]. EMT promotes tumor cell invasion and metastasis to distant organs, which play important roles in tumor progression [20, 21]. EMT program can enhance tumor migration, invasion, and tumor immune evasion [22]. EMT is associated with the cell number of immunosuppressive cells, such as myeloid-derived suppressor cells (MDSCs), and the expression of immune checkpoints, such as PD-L1 [23]. Therefore, researching the molecular mechanism of EMT is necessary to develop new therapeutic strategies to inhibit metastasis, regulate immune microenvironment, and improve the therapeutic effect of ICIs.

Transcription factor YY1 is the key regulator of EMT. YY1 can trigger EMT by upregulating snail expression, which further inhibits E-cadherin expression and increases vimentin expression [8]. Some studies have shown that YY1 plays a regulatory role in normal biological process and has the potential to initiate tumorigenesis. YY1 overexpression or activation is related to uncontrolled cell growth, resistance to apoptosis stimulation, tumorigenesis, and metastasis potential [24–26]. YY1 is involved in the regulation of several developmental immune processes, including B cells and T-cells. T regulatory cell differentiation is directly inhibited by YY1 through the transcriptional inhibition of Foxp3. Cell checkpoint receptors such as PD-1, LAG3, and Tim3 are activated by YY1 transcription, which can lead to T-cells exhaustion and disease progression. YY1 also affects PD-L1 expression through signal crosstalk. Such pathways include p53, STAT3, NF-κB, PI3K/AKT/mTOR, c-Myc, and COX-2. Furthermore, the levels of cytokines and growth factors (e.g., IL6, IL17, TGF-β, and IFN-γ) are affected by YY1 [9]. Therefore, YY1 can serve as a tumor marker for diagnosis and prognosis, and it can be an effective target for antitumor chemotherapy and immunotherapy. However, the mechanism of regulation of YY1 degradation is unclear. In the present study, we found that YY1 interacted with USP7, a member of the deubiquitinating enzyme family. USP7 is a kind of deubiquitinase that plays an important role in cell cycle, cell apoptosis, drug resistance, and other cell activities [27–29]. USP7 can remove ubiquitin tags from specific protein substrates to alter their degradation rate [30]. USP7 targets a variety of proteins such as p53 and Rad18 and regulates cell activities, including DNA damage tolerance, protein recycling, and cell division [31–34]. The current work showed that USP7 could protect YY1 from ubiquitin-mediated degradation and promote the EMT of HCC cells. Analysis of clinical data from TCGA confirmed that USP7 and YY1 expression levels were positively correlated with the pathological grade of HCC, and high USP7 expression suggested poor HCC prognosis. These results suggested that inhibiting USP7-mediated YY1 ubiquitination may be a potential strategy for HCC therapy. Through computer simulation, we selected ISO from about 1000 traditional Chinese medicine monomers as it had the highest docking score with the USP7 active site. ISO is a flavonoid from seabuckthorn fruit and is also a direct metabolite of quercetin. This work showed that ISO could inhibit the deubiquitinase activity of USP7, promote the ubiquitin-mediated degradation of YY1, and inhibit EMT in HCC cells.

EMT can promote the formation of tumor immunosuppression microenvironment. YY1 plays an important regulatory role in tumor cell EMT and tumor immune microenvironment formation. YY1 can also regulate PD-L1 expression. Therefore, the inhibition of YY1-mediated EMT combined with ICIs exerted a synergistic antitumor effect. The PD-L1 protein was encoded by the CD274 gene and is expressed in several types of tumor cells, such as ovarian cancer and HCC. Tumor cells block T cell function and antitumor response by upregulating PD-L1, leading to enhanced tumorigenesis and aggressiveness [35]. In the present study, a drug-development strategy combining the regulation of YY1 expression with the antagonism of the PD-1/PD-L1 pathway was proposed, and the nanodrug HMSN-ISO@ProA-PD-L1 Ab was designed. It can target tumor cells, release ISO, inhibit YY1-mediated malignant progression of tumors, and improve the killing effect of T cells on tumors by inhibiting the PD-1/PD-L1 pathway, exerting a good antitumor effect. To achieve anti-PD-L1 antibody loading, intermediate nanoparticle platforms loaded with Protein A were designed. Protein A is derived from a strain of *Staphylococcus aureus* containing five domains that can specifically bind to the Fc segment of the antibody IgG molecule, whereas no or only very weak binding to other heteroproteins occurred. Therefore, the nanoparticle also provided a platform for loading antibody drugs, which was conducive to the combination of antibody drugs and multiple drugs.

## Conclusions

In conclusion, we revealed for the first time that USP7 interacted with YY1, and that USP7 can stabilize YY1 expression through its deubiquitination activity. ISO can inhibit EMT by inhibiting the deubiquitination activity of USP7 to YY1 in HCC. The nanodrug HMSN-ISO@ProA-PD-L1 Ab can improve tumor immune microenvironment and inhibit YY1-mediated tumor progression. This work also provided a platform for loading antibody drugs by utilizing Protein A.

## Data Availability

The data that support the findings of this study are available from the corresponding author upon reasonable request.

## List of Abbreviations

HCC: Hepatocellular carcinoma
ICIs: Immune checkpoint inhibitors
EMT: Epithelial mesenchymal transition
YY1: Yin-yang-1
ISO: Isorhamnetin
HMSNs: Hollow mesoporous silica nanoparticles
HMSN-ISO@ProA-PD-L1 Ab: Isorhamnetin and anti-PD-L1 antibody dual-functional mesoporous silica nanoparticles
CHX: Cycloheximide
TCGA: The Cancer Genome Atlas
A-HMSNs: Amine-functionalized HMSNs
Co-IP: Co-immunoprecipitation
IHC: Immunohistochemistry
CFSE: Carboxyfluorescein succinimidyl ester
